# Cerebral Amyloid Angiopathy‐Related Inflammation in Iatrogenic Cerebral Amyloid Angiopathy

**DOI:** 10.1111/ene.70198

**Published:** 2025-05-09

**Authors:** Larysa Panteleienko, Dermot Mallon, Cho Me Me Htet, Nathaniel Lizak, Michael Zandi, Gargi Banerjee, David J. Werring

**Affiliations:** ^1^ Stroke Research Centre, Department of Brain Repair and Rehabilitation UCL Queen Square Institute of Neurology London UK; ^2^ Department of Neurology Bogomolets National Medical University Kyiv Ukraine; ^3^ Neuroradiological Academic Unit UCL Queen Square Institute of Neurology London UK; ^4^ Lysholm Department of Neuroradiology National Hospital for Neurology and Neurosurgery London UK; ^5^ National Hospital for Neurology and Neurosurgery Queen Square, University College London Hospitals NHS Foundation Trust London UK; ^6^ Department of Neuroinflammation UCL Queen Square Institute of Neurology London UK; ^7^ MRC Prion Unit at UCL Institute of Prion Diseases London UK

**Keywords:** amyloid‐beta, cadaveric dura mater, cerebral amyloid angiopathy‐related inflammation, iatrogenic cerebral amyloid angiopathy, prion

## Abstract

**Introduction:**

Cerebral amyloid angiopathy (CAA) related inflammation (CAA‐ri) is considered to be a distinct syndrome caused by an inflammatory response to amyloid‐β deposition in the walls of small leptomeningeal and cortical vessels in patients with sporadic CAA. However, recent data suggest that inflammation might contribute to a broader range of CAA subtypes.

**Results:**

We describe a case of probable iatrogenic CAA (iCAA), which manifested with multiple intracerebral haemorrhages complicated by the development of clinical and radiological features of CAA‐ri, which responded to steroids. Clinical, neuroimaging and CSF data suggested possible co‐existing Alzheimer's pathology.

**Discussion:**

CAA‐ri may occur in association with iCAA, suggesting that a broader spectrum of patients might benefit from steroid treatment than previously assumed.

## 1 Introduction

Cerebral amyloid angiopathy (CAA) related inflammation (CAA‐ri) is often considered a distinct subtype of CAA, but recent data challenge this view, suggesting that inflammation may contribute to a range of syndromes associated with CAA [1, 2]. Here, we report a patient with iatrogenic CAA (iCAA) with clinical and radiological features suggesting CAA‐ri.

## 2 Results

### 2.1 Case Description

A 49‐year‐old woman presented after a generalised tonic–clonic seizure with a fall, after reporting progressive forgetfulness in the preceding 3 weeks. Prior to this presentation, she worked as a police administrator, lived alone, and was fully independent.

**FIGURE 1:**
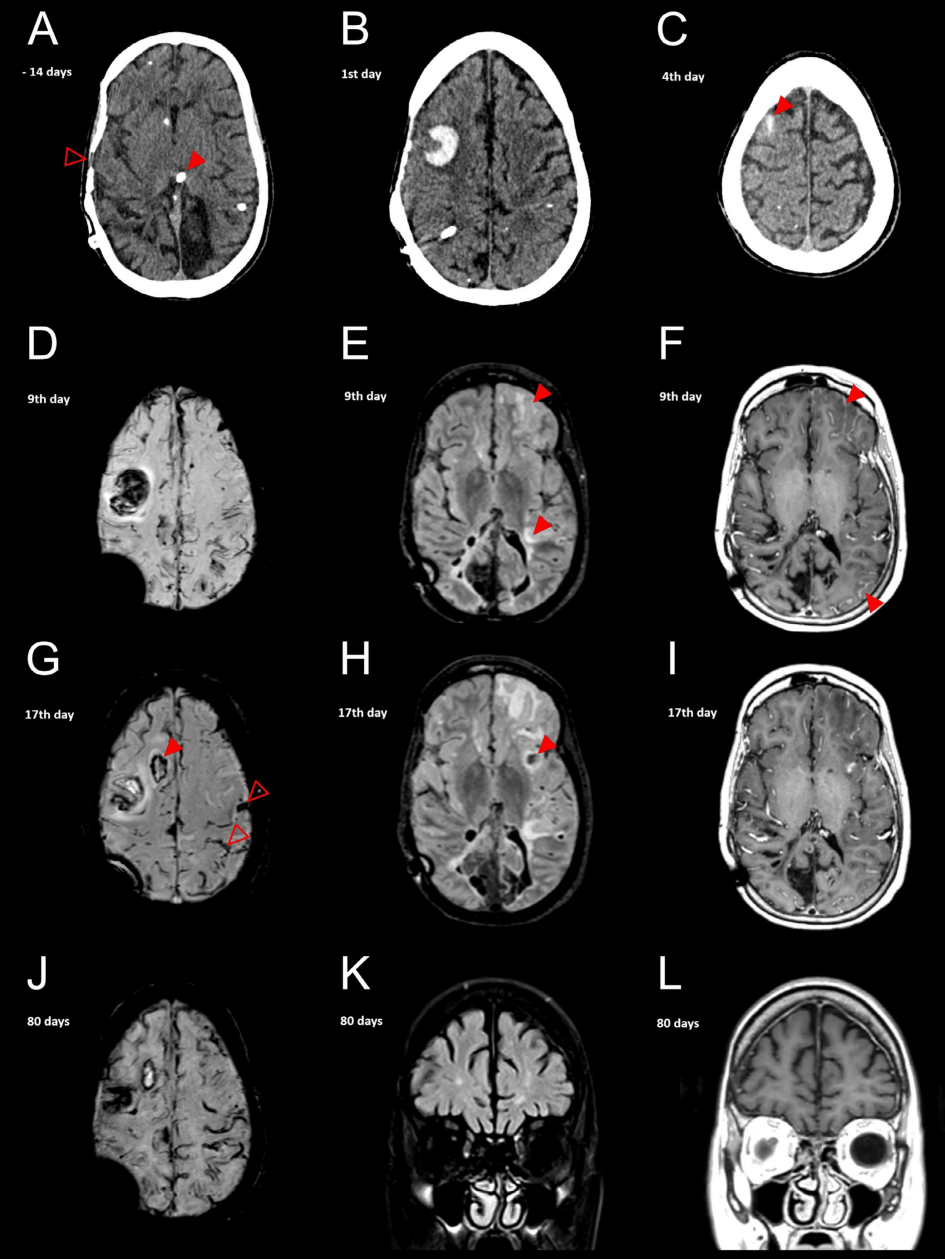
Representative CT and MRI images of the disease progression (with time indicators from hospitalisation due to the described case, days). At first presentation to the Emergency Department, this patient's CT head scan (A) showed no acute findings, but did demonstrate presence of an intraventricular catheter (red arrow), an old right parietal craniotomy (open red arrow), and scattered calcified lesions in both cerebral hemispheres with bilateral occipital white matter volume loss (consistent with her prior history of congenital toxoplasmosis). Two weeks later, when she presented again, her CT head showed an acute intracerebral haemorrhage involving the right middle frontal gyrus (B). Following a further clinical deterioration, her CT scan 3 days later revealed an acute convexity subarachnoid haemorrhage involving the right superior frontal sulcus (C). MRI brain (D), performed 5 days after the acute convexity subarachnoid haemorrhage, showed widespread disseminated cortical superficial siderosis involving both hemispheres and scattered foci of susceptibility artefact, representing lobar microhaemorrhages. T2‐weighted fluid‐attenuated inversion recovery (FLAIR) imaging showed sulcal hyperintensity and mild subcortical vasogenic oedema (arrows) involving the left frontal lobe and near the left temporal stem (E). Post‐gadolinium T1‐weighted imaging showed extensive and prominent leptomeningeal enhancement along the basal surface of the left frontal lobe and over the left parieto‐occipital region (F). Repeat MRI (17 days from admission) showed a further acute convexity subarachnoid haemorrhage overlying the left frontal convexity (open red arrows; G) and two acute intracerebral haemorrhages, involving the right superior frontal gyrus (arrow; G) and the left sub‐insular white matter (arrow; H). Although the previously observed leptomeningeal enhancement had improved (I), there was progression of the left frontal and temporal vasogenic oedema (H). Two months after treatment with steroids, the haematomas had matured, cortical superficial siderosis had progressed (J) and the vasogenic oedema, sulcal hyperintensities (K) and leptomeningeal enhancement (L) had resolved.

CT head (Figure 1A) and blood tests showed no acute changes, and she was subsequently discharged. Two weeks later, she presented with three secondary generalised tonic–clonic seizures and left‐sided weakness. Repeat CT head showed an acute right frontal intracerebral haemorrhage (ICH; Figure 1B).

Five days after admission, the patient developed focal status epilepticus with impaired awareness and weakness of the left arm and leg; 3 days later, she became drowsy and agitated, with gaze deviation to the right. Repeat CT head (Figure 1C) showed a new acute right frontal convexity subarachnoid haemorrhage (cSAH).

During the week following this event, the patient had fluctuating confusion and focal seizures which were treated with a combination of antiseizure medications. MRI brain showed widespread cortical superficial siderosis and lobar microhaemorrhages (Figure 1D), with evidence of sulcal hyperintensities and vasogenic edema in the left frontal lobe (Figure 1E) and leptomeningeal contrast enhancement (Figure 1F).

Her previous medical history included childhood hydrocephalus and bilateral occipital injury secondary to congenital toxoplasmosis managed with a ventriculoperitoneal shunt that required multiple surgical revisions; a review of her childhood medical notes confirmed the use of cadaveric dura (Lyodura) during the last of these shunt revisions (aged 9 years); this history and current presentation with multiple intracranial haemorrhages were consistent with the diagnosis of iCAA [3]. She had well‐managed epilepsy with generalised tonic–clonic seizures, treated with Levetiracetam 1250 mg daily (the last seizure before this presentation was 10 years ago), and severe visual impairment (both since childhood).

The patient continued to experience increasingly frequent seizures despite the treatment escalation. A subsequent MRI revealed further acute cSAH over the left frontal convexity and two new ICH with worsening vasogenic edema (Figure 1G,H), but there was decreased leptomeningeal enhancement (Figure 1I).

Seventeen days into her acute stroke admission, the patient became drowsy in the context of ongoing seizures. She had a lumbar puncture, which showed 80 × 10^6^/L red cells, 4 × 10^6^/L white cells (all lymphocytes), elevated protein (2.21 g/L; normal, 0.13–0.45 g/L), without oligoclonal bands (serum and CSF). Systemic and CSF infective, systemic vasculitis and autoimmune tests were negative. Testing for CSF markers of neurodegeneration showed low amyloid‐β (Aβ) (1–42152 pg/mL, normal, 627–1,322 pg/mL; 1–40 2,824 pg/mL, normal 6,914–22,606 pg/mL; Aβ 1–42/1–40 ratio 0.054, normal ≥ 0.65), elevations of total Tau (1,193 pg/mL; normal 146–595 pg/mL), pTau‐181 (71 pg/mL; normal 0–57 pg/mL) and neurofilament light (> 10,000 pg/mL; normal 0–967 pg/mL).

Given her continued deterioration (20 days after admission), based on the clinical presentation and MRI findings (asymmetric white matter hyperintensities, sulcal hyperintensities, meningeal enhancement and extensive haemorrhagic markers of CAA) she was treated with steroids for a presumed diagnosis of CAA‐related inflammation (CAA‐ri) (intravenous methylprednisolone 1 g daily for 5 days; subsequently, oral prednisolone, 60 mg daily with tapering 5 mg/week). She made some clinical improvement but had three further small left and right frontal lobe ICH in the following 2 weeks.

After 2 months of steroid treatment (80 days into admission), the patient's confusion, agitation and seizures significantly improved and mobility completely recovered. MRI at this stage showed haematomas maturation, resolution of vasogenic oedema, sulcal hyperintensities and leptomeningeal enhancement (Figure 1J–L). Despite this improvement, she had persistent and severe cognitive impairment requiring constant care, and episodic seizures; bilateral medial temporal lobe volume loss on MRI was also noted (not shown). The patient's clinical, neuroimaging and CSF neurodegenerative marker findings raise the possibility of co‐existing Alzheimer's disease. At the time of writing, she remained in hospital (345 days) with a plan for transfer to a nursing home. She had no further intracranial haemorrhages since radiological resolution of CAA‐ri.

## 3 Discussion

The case we describe here is notable for two reasons. Firstly, the patient has early‐onset CAA [4] with prior exposure to cadaveric dura, therefore fulfilling proposed diagnostic criteria for probable iCAA [3, 5]. Secondly, she had both clinical and radiological evidence of CAA‐ri [6], with a complicated clinical course characterised by multiple intracranial haemorrhages and limited improvement only after a prolonged course of steroid treatment. Whilst radiological changes suggestive of inflammation have recently been recognised in iCAA [5], to the best of our knowledge, this is the first detailed description of the clinical and radiological phenotype of a person with iCAA presenting with CAA‐ri, with some apparent limited clinical and radiological improvement and stabilization after steroid treatment. The high frequency of haemorrhagic events in this patient, many of which localised to the right frontal lobe (i.e., spatial and temporal clustering), supports the argument that acute inflammation might contribute to a locally active haemorrhagic vasculopathy in CAA [1].

CAA‐ri typically occurs in older adults and can be diagnosed with a clinical presentation including headaches, cognitive decline, seizures, focal neurological symptoms and distinctive MRI findings showing areas of vasogenic oedema in the brain [6]. The case reported here suggests that CAA‐ri is not necessarily a discrete subtype due to an inflammatory response to sporadic CAA but may also occur in association with iCAA. The clinical implication of our observations is that a broader spectrum of patients with different CAA subtypes might benefit from steroid treatment than has previously been assumed [2]. A recent multicentre cohort study reported various combinations of cortical oedema, parenchymal or sulcal hyperintensities [7], in 14/51 (27.4%) patients with iCAA at any point during a median follow‐up period of 3.7 years. These were mostly transient and reversible, suggesting that a dynamic inflammatory response can occur in some patients with iCAA. We suggest that clinicians consider the possibility of CAA‐ri in patients with all forms of CAA, including iatrogenic and genetic subtypes [8], as early recognition and treatment could improve clinical outcomes.

## Author Contributions

**Larysa Panteleienko:** conceptualization, writing – original draft, methodology, writing – review and editing, data curation, investigation. **Dermot Mallon:** investigation, writing – review and editing, visualization. **Cho Me Me Htet:** writing – review and editing, investigation. **Nathaniel Lizak:** investigation, writing – review and editing. **Michael Zandi:** investigation, writing – review and editing, supervision. **Gargi Banerjee:** conceptualization, writing – original draft, writing – review and editing, data curation. **David J. Werring:** conceptualization, investigation, writing – original draft, writing – review and editing, supervision, methodology.

## Consent

The patient did not have the capacity, so their next of kin kindly gave written consent for publication.

## Conflicts of Interest

The authors declare no conflicts of interest.

## Data Availability

The data that support the findings of this study are available on request from the corresponding author. The data are not publicly available due to privacy or ethical restrictions.
